# Berberine *via* suppression of transient receptor potential vanilloid 4 channel improves vascular stiffness in mice

**DOI:** 10.1111/jcmm.12645

**Published:** 2015-07-14

**Authors:** Jie Wang, Tao Guo, Qi-Sheng Peng, Shou-Wei Yue, Shuang-Xi Wang

**Affiliations:** a The Key Laboratory of Cardiovascular Remodelling & Function Research, Chinese Ministry of Education and Chinese Ministry of Health, Qilu Hospital, Medical School of Shandong UniversityShandong, China; b Department of Physical Medicine & Rehabilitation, Qilu Hospital, Medical School of Shandong UniversityShandong, China; c Key Laboratory for Zoonosis Research, Ministry of Education, Institute of Zoonosis, Jilin UniversityChangchun, China

**Keywords:** berberine, transient receptor potential vanilloid 4, vascular smooth muscle cells, blood pressure, vascular stiffness, ageing

## Abstract

Berberine, as an alkaloid found in many Chinese herbs, improves vascular functions in patients with cardiovascular diseases. We determined the effects of berberine in hypertension and vascular ageing, and elucidated the underlying mechanisms. In isolated aortas, berberine dose-dependently elicited aortic relaxation. In cultured cells, berberine induced the relaxation of vascular smooth muscle cells (VSMCs). Overexpression of transient receptor potential vanilloid 4 (TRPV4) channel by genetic approaches abolished the berberine-induced reduction in intracellular Ca^2+^ concentration in VSMCs and attenuated berberine-elicited vessel dilation in mice aortas. In deoxycorticosterone acetate (DOCA)-induced hypertensive model, treatment of mice with berberine or RN-1734, a pharmacological inhibitor of TRPV4, significantly decreased systemic blood pressure (BP) in control mice or mice infected with an adenovirus vector. However, berberine-induced effects of lowering BP were reversed by overexpressing TRPV4 in mice by infecting with adenovirus. Furthermore, long-term administration of berberine decreased mean BP and pulse BP, increased artery response to vasodilator and reduced vascular collagen content in aged mice deficient in apolipoprotein E (Apoe-KO), but not in Apoe-KO old mice with lentivirus-mediated overexpression of TRPV4 channel. In conclusion, berberine induces direct vasorelaxation to lower BP and reduces vascular stiffness in aged mice through suppression of TRPV4.

## Introduction

Berberine, an isoquinoline alkaloid originally isolated from the Chinese herb *Coptis chinensis*, is an anti-microbial drug routinely prescribed for the treatment of diarrhoea in many Asian countries 1. In this form it is reported to exert anti-fungal, anti-bacterial/viral and anti-oncogenic effects, as well as a beneficial effect on diabetes, atherosclerosis and hyperlipidemia 2,3. Although vasorelaxant effects of berberine have been observed in different animal models 4, the underlying mechanism and the effects of berberine on hypertension and vascular stiffness, which are associated with elevated vascular tone, are poorly understood.

Vascular smooth muscle cells (VSMCs) contraction is predominantly regulated by myosin light chain (MLC), which is determined by increased intracellular Ca^2+^ ([Ca^2+^]_i_) concentration through calmodulin (CaM)-dependent MLC kinase, resulting in phosphorylation of MLC at serine 19 and consequent contraction 5. The Ca^2+^ influx is a common mechanism of transient increase in the cytoplasmic free Ca^2+^ concentration triggered by cell depolarization 6. This form of Ca^2+^ signalling activates essential cellular processes including cardiac contraction, regulation of a smooth muscle tone, *etc*. Whether and how berberine reduces Ca^2+^ influx in VSMCs still remains unknown.

Transient receptor potential vanilloid 4 (TRPV4) is a Ca^2+^-permeable cation channel, originally identified as a transducer of hypotonic stimuli 7. TRPV4 can be activated by various physical and chemical stimuli. Studies have reported that berberine produced inhibitory effects on Ca^2+^ channels 8. Thus, we suggested that berberine *via* suppression of TRPV4 might lower blood pressure (BP) by inhibiting vessel contraction or eliciting vasorelaxation. Our data in this study suggest that berberine induces endothelium-independent relaxations in VSMCs to lower BP and to delay vascular stiffness by suppressing TRPV4 and the associated Ca^2+^ signalling in mice.

## Materials and methods

### Animals

Wild-type (WT, C57B16) mice and gene knockout of AMP-activated protein kinase (AMPKα1-KO), AMPKα2 (AMPKα2-KO), endothelial nitric oxide synthase (eNOS-KO) and apolipoprotein E (Apoe-KO) mice, 8–12 weeks of age, 20–25 g, were obtained from the Jackson Laboratory (Bar Harbor, ME, USA). Mice were housed in temperature-controlled cages with a 12-hr light–dark cycle and given free access to water chows. The animal protocol was reviewed and approved by the University of Shandong University, Institute of Animal Care and Use Committee and the local ethics committee, in accordance with the Helsinki Declaration.

### Measurement of tension development in aortic rings

*In vivo* or *ex vivo* organ chamber study was performed as described previously 9–12. In short, descending aorta was cut into rings (3–4 mm in length) and suspended and mounted to organ chamber filled with Kreb's buffer, gassed with 95% O_2_ plus 5% CO_2_. The contractile response was elicited by phenylephrine (PE), U46619 or KCl. Accumulative berberine, sodium nitroprusside (SNP) or phentolamine mesylate was added into the organ bath to induce vessel relaxation.

### Blood pressure measurement

Blood pressure was determined by invasive left carotid catheter or radiotelemetry methods as described previously 13. For invasive left carotid catheter, a catheter was inserted into the left common carotid artery. Blood was directed to a pressure transducer through the catheter to obtain computerized BP measurements (AD Instruments, Bella Vista, New South Wales, Australia). Blood pressure signals were recorded and analysed using a software of powerlab system (Lab chart 5.0, Bella Vista, New South Wales, Australia). Radiotelemetry method of surgical procedure about insertion of radiotelemetry transmitter has been described above 13.

### DOCA-salt hypertensive mice

DOCA-salt hypertension was created as previously described 14. 150 mg/kg DOCA were implanted subcutaneously in mice given water containing 1.0% NaCl and 0.2% KCl.

A full description of materials and methods used, including generation of virus vector, cell culture, adenovirus infection to cells, measurement of [Ca^2+^]_i_ concentration, western blot analysis, measurement of tension development in aortic rings, induction of hypertension by DOCA-salt in mice, BP measurement, picrosirius red staining, protocol for *in vivo* animal experiments in details and statistical analysis can be found in the Data S1.

## Results

### Berberine dose-dependently induces direct vessel relaxation in isolated mice aortas

**Figure 1:**
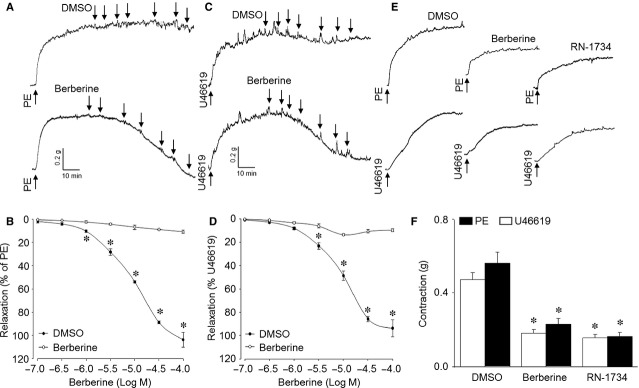
Berberine inhibits agonists-induced contraction and induces aortic relaxation in isolated mice aortas. (**A**–**D**) The contraction of isolated aortic ring in organ chamber was induced by PE (1 μM) or U46619 (30 nM). Berberine was added in to the organ bath as indicated dose when the contraction was stable. (**A**) The representative tracing of berberine-induced relaxation in PE-precontracted aorta ring. (**B**) Summary data for berberine-induced relaxation in **A**. (**C**) The representative tracing of berberine-induced relaxation in U46619-prechallenged aorta ring. (**D**) Summary data for berberine-induced relaxation in **C**. All quantitative results in **B** and **D** are expressed as mean ± SEM 6 mice were in each group. *P < 0.05 *versus *DMSO. (**E** and **F**) Isolated aortic ring was incubated with berberine (10 μM) or RN-1734 (10 μM) for 60 min. followed by stimulation with U46619 (30 nM) or PE (1 μM). The contraction of aortic ring was recorded. (**E**) The representative tracing of aortic ring contraction. (**F**) Summary data for the effects of berberine on agonists-induced contraction. Quantitative results are expressed as mean ± SEM 6 mice were in each group. *P < 0.05 *versus* DMSO.

We first determined the effects of berberine on vessel relaxation by organ chamber study. As shown in Figure1A and B, aortic rings were pre-contracted by PE (1 μM), which induces vessel contraction through activation of G protein-coupled receptor (α-adrenoreceptor). When the contraction reached the peak and kept in stable state, accumulative berberine (0.1–100 μM) was added into an organ bath to induce vessel relaxation. Berberine, but not Dimethyl Sulphoxide (DMSO), started to relax aortic rings at 1 μM and completely reversed PE-induced contraction at 0.03 mM. In addition, berberine also dose-dependently induced vessel relaxation in aortic rings pre-contracted by U46619 at 30 nM (Fig.1C and D), which is another activator of G protein-coupled receptor (thromboxane-receptor). Besides, the dose–response curves of berberine in PE- and U46619-contracted aortic rings were fitted by the Hill equation (Fig.1B and D). The IC50 values of berberine on vessel relaxation were also calculated by fitting Hill equation to 6 individual data sets obtained from each aortic ring. The IC50s of berberine to induce relaxation in PE and U46619 are 8.93 ± 0.58 μM and 9.47 ± 0.62 μM, which are similar (*P* > 0.05), indicating berberine induces vessel relaxation in aortic arteries pre-contracted with multiple agonists.

### Berberine suppresses the contractions of isolated mice aortas induced by agonists

We then investigated whether berberine suppressed vessel constriction in isolated mice aortic rings. Prior to induction of aortic contraction, isolated aortic rings from mice were incubated with berberine (10 μM) or DMSO for 60 min. As shown in Figure1E and F, incubation of aortic rings with berberine but not DMSO significantly suppressed vessel contraction induced by PE and U46619, suggesting that berberine also has the function to attenuate vessel constriction.

### Berberine-induced relaxation is endothelium-independent

To examine whether the vasodilation induced by berberine is mediated by endothelium, we detected the effects of berberine on vascular relaxation on aortic rings by removing the endothelium or by the incubation of aortas with eNOS inhibitor NG-Nitro-L-arginine Methyl Ester (L-NAME, 1 mM, 30 min.). As shown in Figure S1A and B, berberine dose-dependently induced vessel relaxation in endothelium-denuded aortas or L-NAME-pretreated aortas, suggesting that berberine-induced relaxation is independent of endothelium (or eNOS). This viewpoint was further confirmed by using aortas from eNOS gene knockout mice (eNOS-KO). Similar to the removal of endothelium or L-NAME in aortas, deletion of eNOS did not alter berberine-induced vasorelaxation (Fig. S1A and B), providing further evidence of berberine-induced endothelium-independent relaxation.

### AMPK is not involved in berberine-induced relaxation

In addition, berberine can activate AMPK in vascular wall cells 15,16. We determined whether berberine elicits relaxation on aortic rings if AMPK is inhibited. Unexpectedly, inhibition of AMPK by compound C (10 μM, 30 min.), which is a well-known AMPK inhibitor 17, did not abolish berberine-induced relaxation (Fig. S1C and D).

To exclude the possibility that these results were because of non-specific effects of compound C treatment, we repeated these experiments with mice aortas from AMPK α1 or α2 subunits gene knockout mice (AMPKα1-KO, AMPKα2-KO). As shown in Figure S1C and D, ablation of AMPK α1 or α2 did not change the effects of relaxation induced by berberine in WT mice aortas.

### Berberine inhibits PE-induced MLC phosphorylations and contractions in cultured VSMCs

**Figure 2:**
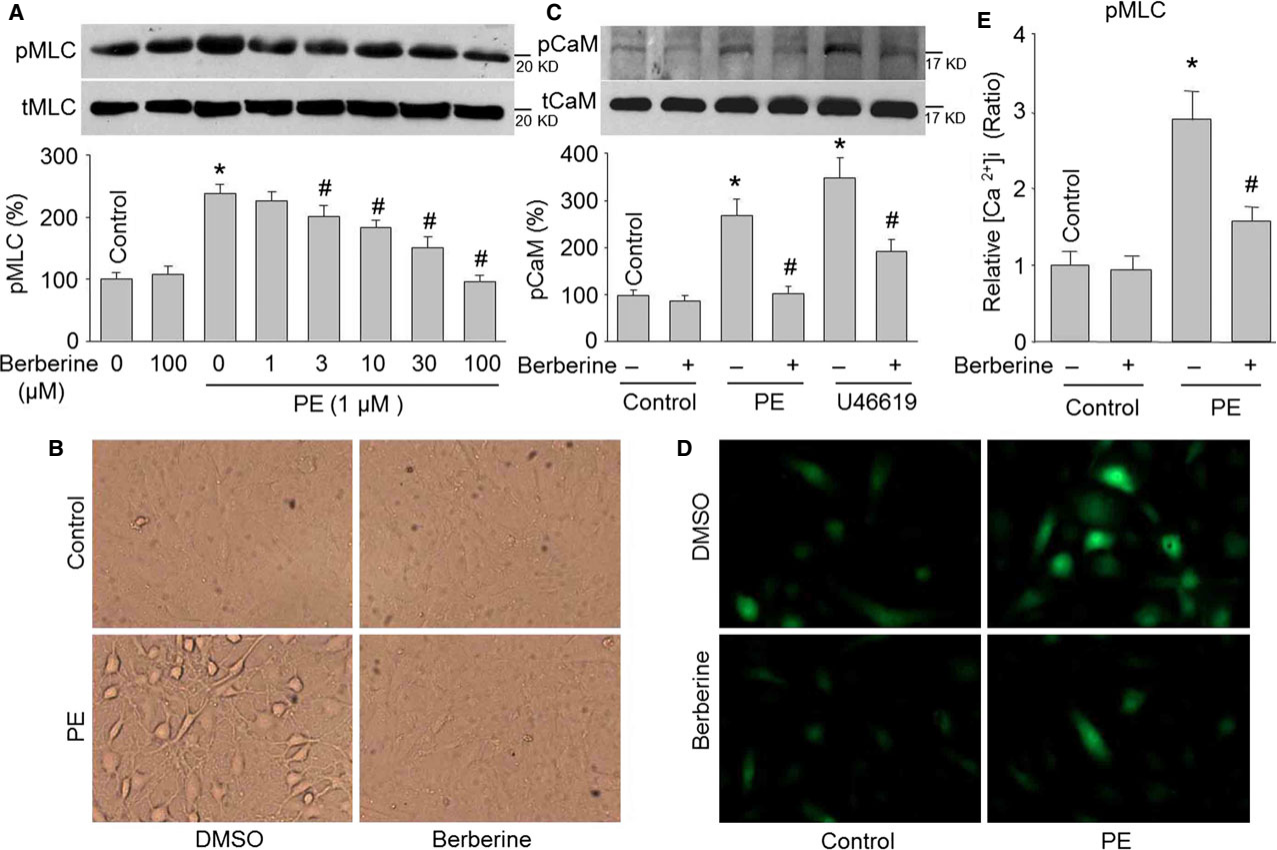
Berberine reduces intracellular Ca^2+^ concentration and induces direct relaxation in cultured human vascular smooth muscle cells (VSMCs). (**A**) Cultured human VCMCs were incubated with berberine as indicated concentration (1–100 μM) for 60 min. and then stimulated with PE (1 μM) for 30 min. Total cell lysates were subjected to perform Western blot for detection of pMLC levels. The MW of pMLC (or tMLC) is about 20 kD, which matches the expected sizes. The blot is a representative blot obtained from three independent experiments. Quantitative results are expressed as mean ± SEM, *P < 0.05 *versus* Control, ^#^*P* < 0.05 *versus *PE alone. (**B**) Cultured human VCMCs were incubated with berberine (10 μM) for 60 min. and then stimulated with PE (1 μM) for 30 min. The representative picture of cell morphology was taken by the microscope from three independent experiments. (**C**) Cultured human VCMCs were incubated with berberine (10 μM) for 30 min. and then stimulated with PE (1 μM) or U46619 (30 nM) for 30 min. Total cell lysates were subjected to perform Western blot for detection of pCaM levels. The MW of pCaM (or tCaM) is about 17 kD, which matches the expected sizes. The blot is a representative blot obtained from three independent experiments. Quantitative results are expressed as mean ± SEM, *P < 0.05 *versus* Control, ^#^*P* < 0.05 *versus *PE or U46619 alone. (**D**) Cultured human VCMCs were incubated with berberine (10 μM) for 60 min. and then stimulated with PE (1 μM) for 30 min. Intracellular Ca^2+^ ([Ca^2+^]_i_) concentration was assayed by detecting Fluo-4-AM fluorescence. Relative [Ca^2+^]_i_ concentration was calculated by the ratio of fluorescent intensity to control. The fluorescent intensity in control was set up as 1. The representative picture was presented from three independent experiments. (**E**) Summary data for [Ca^2+^]_i_ concentration in **D**. Quantitative results are expressed as mean ± SEM, *P < 0.05 *versus* Control, ^#^*P* < 0.05 *versus *PE alone.

The contractile state of a VSMC induced by agonist depends on the phosphorylation of MLC at serine 19 5,18. Thus, we assayed the effects of berberine on MLC phosphorylation by Western blot. As shown in Figure2A, treatment of cultured VSMCs with PE (1 μM) for 30 min. dramatically increased MLC phosphorylation at serine 19 but not enhanced total MLC protein level. However, pre-treatment of VSMCs with berberine (1–100 μM, 5 min.) dose-dependently inhibited PE-induced MLC phosphorylation.

Further, we detected the alteration of cell morphology in berberine-pretreated PE-stimulated VSMCs in an *in vitro* cell culture model 19. A 30-min. exposure of confluent VSMCs to PE (1 μM) resulted in spiderlike cell morphology (Fig.2B). The contracted cells had narrow flaps around the round cell body. The perimeter and the area of contracted cells were decreased.

### Berberine reduces intracellular Ca^2+^ signalling in VSMCs activated by agonists

We suggested that berberine *via* reduction of [Ca^2+^]_i_ induces relaxation of VSMCs. To test this idea, we detected Ca^2+^ signalling by assaying [Ca^2+^]_i_ level and CaM phosphorylation at serine 81, which represents its activity 20. In Figure2C, both PE and U46619 dramatically increased CaM serine 81 phosphorylation without altering total protein levels of CaM. Pre-incubation of VSMCs with berberine (10 μM) for 30 min. reduced CaM phosphorylation. As suggested , the [Ca^2+^]_i_ level was significantly up-regulated by PE treatment assayed by measuring fluo-4/AM fluorescence (Fig.2D and E). The PE-increased [Ca^2+^]_i_ level is berberine-reversible.

### Berberine-reduced VSMC contraction is VOCC-independent

A derivative of berberine, CPU86017 (as structured in Fig. S2), has been reported to perform as a blockade of voltage-operated calcium channel (VOCC) in myocardium 21. Thus, we examined whether berberine also functions as an antagonist of VOCC to reduce [Ca^2+^]_i_ levels by using KCl, which induces VSMC contraction, as a result of membrane depolarization by causing Ca^2+^ entry through VOCC. As indicated in Figure S3A, 30-min. exposure of confluent VSMCs to KCl (60 mM) resulted in significant cell contractions. Unexpectedly, pre-incubation of VSMCs with berberine did not reverse the KCl-induced VSMC contractions.

We further confirmed the effects of berberine on KCl-induced vessel constriction in isolated mice aortas. Prior to induction of aortic contraction, isolated aortic rings from mice were incubated with berberine (10 μM) or DMSO for 60 min. As shown in Figure S3B and C, incubation of aortic rings with either berberine or DMSO did not alter KCl-induced vessel contraction. Differently, berberine, which induced vasodilation in PE- or U46619-prechallneged vessel (Fig.1A and B), failed to induce vasorelaxation in KCl-prechallenged aortic rings (Fig. S3D and E). Taking these data together, it suggests that berberine performs its function as a vasodilator, which is VOCC-independent.

### Pharmacological activation of TRPV4 abolishes berberine-suppressed VSMC contractions in VSMCs

The TRPV4 channel is a regulator of intracellular Ca^2+^ in almost all cells which regulates vascular tone and BP 22–24. Thus, we examined whether berberine functions as an antagonist of TRVP4 to reduce the [Ca^2+^]_i_ level. We used the known TRPV4 agonists (4α-PDD, GSK1016790A) or antagonists (RN-1734) to activate or inhibit TRPV4, of which the chemical structures are shown in Figure S2. Incubation of VSMCs with berberine or RN-1734 significantly reversed the morphological alteration of cultured VSMCs from resting to contractile (Fig. S4A, c and d) or PE/U46619-induced contraction in aortic rings (Fig.1E and F). However, berberine was unable to reverse PE-induced VSMC contractions in presence of 4α-PDD or GSK1016790A (Fig. S4A, e and f). Berberine derivative of CPU86017 also induced VSMCs relaxation if TRPV4 was not activated (Fig. S4A, g and h), indicating that TRPV4 might be a pharmacological target of berberine to suppress VSMC contractions.

### Overexpression of TRPV4 abolishes berberine-induced reduction of intracellular Ca^2+^ concentration in VSMCs

**Figure 3:**
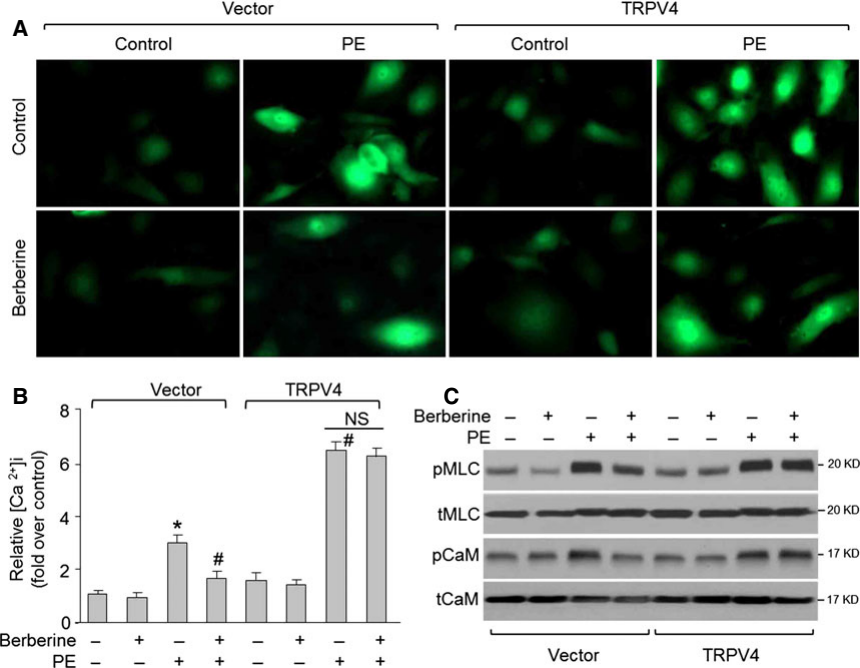
Berberine *via* suppression of transient receptor potential vanilloid 4 (TRPV4) induces relaxation of vascular smooth muscle cells. Cultured human VCMCs infected with adenovirus containing vector or TRPV4 cDNA for 48 hrs were incubated with berberine (10 μM) for 60 min. followed by stimulation with PE (1 μM) for 30 min. (**A**) Intracellular Ca^2+^ ([Ca^2+^]_i_) concentration was assayed by detecting Fluo-4-AM fluorescence. Relative [Ca^2+^]_i_ concentration was calculated by the ratio of fluorescent intensity to control. The fluorescent intensity in control was set up as 1. The representative picture was presented from three independent experiments. (**B**) Summary data for [Ca^2+^]_i_ concentration in **A**. Quantitative results are expressed as mean ± SEM, *P < 0.05 *versus* Vector, ^#^*P* < 0.05 *versus* Vector plus PE. NS indicates no significant difference. (**C**) Total cell lysates were subjected to perform Western blot for detection of pCaM, tCaM, pMLC and tMLC protein levels. The MWs of pMLC (or tMLC) and pCaM (or tCaM) match the expected sizes. The blot is a representative blot obtained from three independent experiments.

We infected VSMCs with adenovirus containing TRPV4 cDNA to confirm whether berberine *via* suppression of TRPV4 induces VSMC relaxation. As shown in Figure S5A, 48-hr adenovirus infection dramatically increased TRPV4 protein expression in cell membrane. PE dramatically increased [Ca^2+^]_i_ levels in VSMCs infected with both vector and TRPV4 as indicated by fluo-4/AM fluorescence (Fig.3A and B). Although berberine still effectively reduced PE-induced enhancement of [Ca^2+^]_i_ levels in vector-infected VSMCs, it failed to suppress the up-regulation of [Ca^2+^]_i_ levels in PE-stimulated VSMCs when TRPV4 was overexpressed. Besides, overexpression of TRPV4 bypasses berberine-inhibited CaM and MLC phosphorylations (Fig.3C) and consequent VSMC contraction (Fig. S4B). These results demonstrate that berberine *via* suppression of TRPV4 reduces [Ca^2+^]_i_ signalling to relax VSMCs.

### Berberine *via* suppression of TRPV4 inhibits vessel contraction and induces vasorelaxation in isolated mice aortas

**Figure 4:**
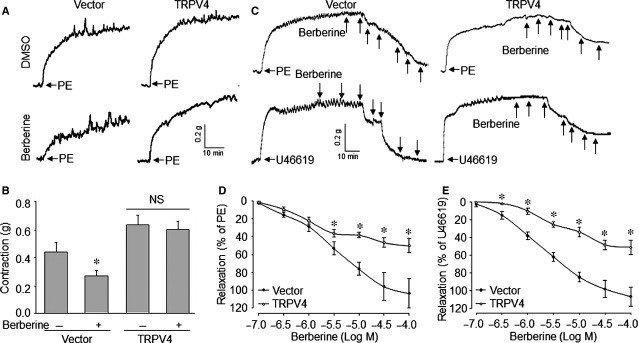
Overexpression of TRPV4 inhibits berberine-induced relaxation in mice aortas. Aortas from mice infected with adenovirus of containing vector or TRPV4 cDNA for 4 weeks were cut into rings and were mounted in organ chamber to detect vessel bioactivity. (**A**) Aortic ring was incubated with berberine (10 μM) for 60 min. followed by stimulation with PE (1 μM). The contraction of aortic ring was recorded by a computer. The representative tracing of aortic ring contraction was shown from six mice. (**B**) Summary data for the effects of berberine on aortic contraction in **A**. Quantitative results are expressed as mean ± SEM,* N* is 6 mice in each group. *P < 0.05 *versus* Vector alone. NS indicates no significant difference. (**C**) The contraction of aortic ring in organ chamber was induced by PE (1 μM) or U46619 (30 nM). Berberine was added into organ bath as indicated dose when the contraction was in the peak. The representative tracing of berberine-induced relaxation on aortic ring was shown from six mice. (**D**) Summary data for berberine-induced relaxation in PE-stimulated aorta in **C**. (**E**) Summary data for berberine-induced relaxation in U46619-stimulated aorta in **C**. Quantitative results are expressed as mean ± SEM 6 mice were in each group. *P < 0.05 *versus* Vector.

Next we detected the effects of overexpression of TRPV4 on berberine-reduced contraction on mice aortas. Mice were infected with adenovirus to overexpress TRPV4 in aortas. As shown in Figure4A and B, incubation of aortic rings with berberine dramatically suppressed PE-induced contractions in aortas from mice infected with the vector. However, berberine did not inhibit PE-induced vasoconstriction in aortas from mice when TRPV4 was up-regulated. Similar to suppression on vessel contraction, berberine elicited direct relaxation in PE/U46619-challenged aortic rings isolated from mice infected with vector (Fig.4C–E). However, these effects of berberine on vasodilation were attenuated by up-regulation of TRPV4.

### Berberine lowers DOCA-salt-induced systemic hypertension in mice

**Figure 5:**
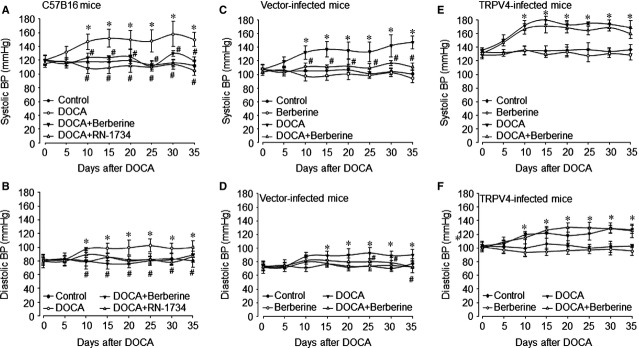
Administration of berberine *via* inhibition of TRPV4 reduces DOCA-induced hypertension in mice. (**A** and **B**) C57B16 mice at the age of 8–12 weeks old were fed with normal diet containing berberine (100 mg/kg/day) and RN-1734 (30 mg/kg/day) for 7 days prior to DOCA infusion. The hypertensive model was induced by DOCA (150 mg/kg) implantation. Blood pressures (BP) and heart rates were monitored by telemetry, as described in Materials and Methods. Systolic BP in **A** and diastolic BP in **B** were analysed. Quantitative results are expressed as mean ± SEM 10–15 mice in each group. *P < 0.05 *versus* Control mice. ^#^*P* < 0.05 *versus *DOCA-implanted mice. (**C** and **D**) C57B16 mice at the age of 8-12 weeks were injected with adenovirus vector via tail vein. 2 weeks later, berberine (100 mg/kg/day) were provided in the diet followed by DOCA implantation for 35 days. Systolic BP in **C** and diastolic BP in **D** in adenovirus-vector-infected mice were analysed. Quantitative results are expressed as mean ± SEM 10–15 mice in each group. *P < 0.05 *versus* Mice infected with vector. ^#^*P* < 0.05 *versus *DOCA-implanted mice infected with vector. (**E** and **F**) C57B16 mice were received infection of adenovirus containing TRPV4 cDNA by tail vein injection followed by administration of berberine (100 mg/kg/day) and DOCA (150 mg/kg) implantation. Systolic BP in **E** and diastolic BP in **F** in mice infected adenovirus containing TRPV4 cDNA were analysed. Quantitative results are expressed as mean ± SEM 10–15 mice in each group. *P < 0.05 *versus* Mice infected with TRPV4 cDNA.

We next tested the effects of berberine on DOCA-salt-induced hypertension. Wild-type (C57B16) mice were implanted with DOCA-salt to establish hypertensive model. Systemic BP was measured by radiotelemetry method. As shown in Figure5A and B, both systolic BP and diastolic BP were increased to the high level at the 10th day and were stable upto the 35th day after DOCA-salt treatment. Administration of mice with berberine completely suppressed DOCA-salt-induced increases of systolic BP and diastolic BP. The effects of berberine on lowering BP were mimicked by a recognized TRPV4 antagonist, RN-1734, indicating that berberine may function as TRPV4 antagonist to lower salt-sensitive BP.

### Overexpression of TRPV4 abolishes the berberine-induced effects of lowering BP in DOCA-salt-treated mice

To further confirm berberine *via* suppression TRPV4 decreases BP, we generated TRPV4-overexpressed mice by infecting WT mice with adenovirus (Fig. S5B). Similar to control C57B16 mice without any infections (Fig.5A and B), berberine also produced significantly BP-lowering effects in mice infected with adenovirus containing vector when implanted with DOCA-salt (Fig.5C and D). As expected, while TRPV4 was up-regulated by adenovirus infection in mice, the increased systolic BP and diastolic BP were not inhibited significantly by berberine (Fig.5E and F). These data demonstrate that the TRPV4 is involved in berberine-induced effects on lowering BP.

### Long-term administration of berberine, through suppression of TRPV4, decreases pulse BP in aged mice

**Figure 6:**
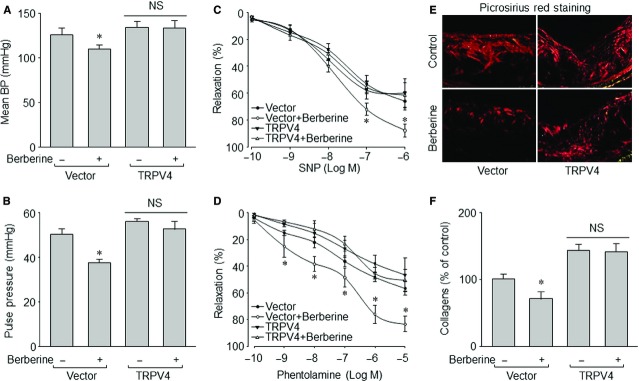
Long-term administration of berberine *via* suppression of TRPV4 delays vascular stiffness in aged Apoe-KO mice. Male Apoe-KO mice at the age of 4 months old received lentivirus infection containing vector or TRPV4 cDNA by tail vein injection once every 2 months. Berberine (50 mg/kg/day) were provided in the high-fat diet starting from the end of 2nd month after lentivirus infection to the end of experiments. The total administrating time of berberine was 12 months. Blood pressures (BP) was monitored by telemetry, as described in Materials and methods. At the end of experiments, all mice were killed under anaesthesia. (**A**) Mean BP. (**B**) Pulse BP. (**C**) SNP-induced vessel relaxation was determined by organ chamber in descending aortas. (**D**) Phentolamine-induced vessel relaxation was assayed by organ chamber in descending aortas. (**E**) Picrosirius red staining in abdominal aorta was performed to detect collagens in vessel wall. (**F**) Summary data for the content of collagen in **E**. All quantitative results are expressed as mean ± SEM 10–15 mice in each group. *P < 0.05 *versus* Mice infected with vector. NS indicates no significant difference.

To investigate whether berberine *via* suppression of TRPV4 improves vessel elasticity in old mice, we generated TRPV4 overexpressed mice by infecting high-fat-diet-fed Apoe-KO mice with lentivirus (Fig. S5C). As depicted in Figure6A and B, 1-year administration of berberine in Apoe-KO mice fed with high-fat diet obviously decreased mean BP and pulse BP (131 ± 17 *versus* 114 ± 12 mmHg for mean BP, *P* < 0.05; 55 ± 7 *versus* 37 ± 4 mmHg for pulse BP, *P* < 0.05) when mice were infected with lentivirus vector alone. However, long-term berberine administration did not lower mean BP and pulse BP (140 ± 23 *versus* 137 ± 18 mmHg for mean BP, *P* > 0.05; 56 ± 9 *versus* 51 ± 6 mmHg for pulse BP, *P* > 0.05) if mice were infected with lentivirus expressing TRPV4. These findings indicate that berberine delays vascular stiffness *via* suppression of TRPV4 in aged mice.

### Up-regulation of TRPV4 inhibits artery response to vasodilators in berberine-treated aged mice

It is worthy of assaying bioactive response of artery to SNP and phentolamine, which are well-used drugs in hypertensive patients. As indicated in Figure6C and D, SNP- or phentolamine-induced dose-dependent relaxation in abdominal aortic ring were improved dramatically in lentivirus-vector-infected mice with 1-year administration of berberine, compared with mice without berberine treatment. However, berberine did not improve vasorelaxation induced by SNP or phentolamine in mice infected with lentivirus expressing TRPV4. Collectively, it suggests that berberine improves vessel bioactivity in aged mice, which is mediated by inhibition of TRPV4.

### Berberine *via* inhibition of TRPV4 reduces collagen contents in artery wall in aged mice

Pathologically, accumulative collagen deposition in artery wall is a character of vascular stiffness 25,26. We finally determined the collagen contents in artery by picrosirius red staining. Similarly, when TRPV4 was up-regulated in Apoe-KO mice, the collagen contents were not reduced significantly by berberine, but it was decreased in mice without overexpression of TRPV4 (Fig.6E and F). These data demonstrate that the TRPV4 is also involved in berberine-mediated effects on collagen deposition in the artery wall of aged mice.

## Discussion

In this study, we provide the first evidence that administration of berberine *in vivo* lowers high BP in DOCA-induced hypertensive mice and reduces vascular stiffness in aged Apoe-KO mice. Mechanically, these effects of berberine are attributable to suppression of TRPV4, decreased [Ca^2+^]_i_ levels, decreased CaM/MLC activity and consequent relaxations in VSMC. In this way, berberine functions as a calcium channel blocker to decreases high BP and vascular hardness.

The major finding of this study is that berberine suppresses DOCA-induced hypertension and ageing-induced vascular stiffness. Physiologically, hypertension and vascular stiffness is related to vascular flexibility and compliance. Previous studies have reported that berberine or its derivative blocked Ca^2+^ influx as a blockade of L-type Ca^2+^ channel in myocardium to treat heart failure 8,27. In the present study, we further identified that TRPV4 as a novel target of berberine to regulate vascular tone through direct relaxation on VSMCs by suppressing Ca^2+^ entry. Accordingly, *in vitro* or *in vivo* overexpression of TRPV4 abolished reduction in [Ca^2+^]_i_ levels in berberine-treated cells and relaxation in isolated aortas from mice feed with berberine. Thus, we reason the exact mechanism of berberine blocking TRPV4 is possibly related to the bind of berberine to the pore core of TRPV4, which is the core of Ca^2+^ channel 28, to misconduct Ca^2+^ current (Fig. S6), similar to RN-1734 does. As a shortcoming of this study, we can only speculate berberine can directly target on TRPV4. Solid evidence should be provided to be sure this possibility.

In general, the function of TRPV4 on vascular endothelial cell in pulmonary artery was sure to induce endothelium-dependent relaxation, which is sensitive to high salt 22,23,29–31. The functions of TRPV4 in other cells or other arteries are more controversial. In this study, we found that RN-1734, a TRPV4 antagonist, significantly inhibited PE/U46619-induced contraction in aortic rings (Fig.1E and F), indicating that activation of TRPV4 was involved in the PE/U46619-induced vessel contraction. For example, Earley *et al*. reported that TRPV4 activation *via* signalling coupling to ryanodine receptors and BKca channels causes vascular smooth muscle hyperpolarization and relaxation in rat cerebral artery 32, inconsistent with our observations. We thought that this discrepancy is because of the different arteries used in our and their studies. Moreover, it is unclear whether and how PE and U46619 can induce TRPV4 activation, which can both activate their specific receptors to induce contraction of VSMC by increasing intracellular Ca^2+^ concentration 18. TRPV4, as a Ca^2+^ permeable non-selective cationic channel, is activated by stimuli including physical, thermal and chemical stimuli, cell swelling, shear stress in particular cells. Importantly, intracellular Ca^2+^, depending on the concentration, either potentiates or inhibits the TRPV4 channel activity 22,28. Thus, we speculate that PE and U46619 can induce TRPV4 activation through elevated intracellular Ca^2+^ concentration.

A major limitation of this study is that the range of berberine to induce vasorelaxation is 10–30 μM. Although other groups have also reported that berberine enhances the endothelium-dependent vasorelaxation and endothelium-independent VSMC dilatation or inhibits the endothelium-independent contraction induced by an agonist at similar concentration 33,34, such concentration of berberine action should not be limited to the Ca^2+^-permeable channel. Membrane penetration and diffusion rate should be analysed to determine the pharmaceutical kinetics of berberine in VSMC. Besides, it has been reported 35 that the K^+^ channel blockers significantly attenuated berberine-induced vasodilatation in the endothelium-denuded arteries, indicating the role of K^+^ channel in berberine-induced VSMC contraction. Further study should focus on the effects of K^+^ channel in VSMC.

In summary, we are the first to report that berberine may suppress TRPV4, decrease [Ca^2+^]_i_ levels and CaM/MLC activity, and induce consequent relaxation in VSMC (Fig. S7). This leads to the anti-hypertensive and anti-vascular ageing effects of berberine in mice. Our discovery of characterization of berberine as an antagonist of the TRPV4 channel would help in the elucidation of TRPV4 function and considered it as a clinical drug to target TRPV4 in treating the high-tension of vascular tone-related cardiovascular diseases, such as hypertension, vascular stiffness and stroke.
