# Supplementary material for: Berberine via suppression of transient receptor potential vanilloid 4 channel improves vascular stiffness in mice
Source: J Cell Mol Med. 2015 Jul 14;19(11):2607–16. doi: 10.1111/jcmm.12645 (PMC4627566; doi:10.1111/jcmm.12645)
Supplement: Supplementary file 1 — Data S1 Supplementary Materials and Methods. [file jcmm0019-2607-sd1.pdf]

Supplementary Materials for

Berberine via Suppression of Transient Receptor Potential Vanilloid 4 Channel Improves Vascular Stiffness in Mice

Jie Wang[1,2], Tao Guo[1], Qi-Sheng Peng[3], Shou-Wei Yue[2], Shuang-Xi Wang[1]

[1]The Key Laboratory of Cardiovascular Remodelling & Function Research, Chinese Ministry of Education and Chinese Ministry of Health, Qilu Hospital, Medical School of Shandong University, China; [2]Department of Physical Medicine & Rehabilitation, Qilu Hospital, Medical School of Shandong University, China; [3]Key Laboratory for Zoonosis Research, Ministry of Education, Institute of Zoonosis, Jilin University, Changchun, China.

Correspondence to Pro. Shuang-Xi Wang, the Key Laboratory of Cardiovascular Remodeling & Function Research, Qilu Hospital Shandong University, No. 107 West Culture Road, Jinan City, Shandong, China, 250012. Tel. 86-531-82169240, Fax 86-531-82169259, Email shuangxiwang@sdu.edu.cn

Contents

1. Supplementary Materials and Methods
2. Supplementary Figure Legends S1-S7

Supplementary Materials and Methods

Animals

Wild-type (WT, C57B16) mice and gene knockout of (AMPKα1-KO), AMPKα2 (AMPKα2-KO), eNOS (eNOS-KO),and apolipoprotein E (Apoe-KO) mice, 8-12 weeks of age, 20-25 g, were obtained from the Jackson Laboratory (Bar Harbor, ME). Mice were housed in temperature-controlled cages with a 12-h light-dark cycle and given free access to water chows. The animal protocol was reviewed and approved by the University of Shandong University, Institute of Animal Care and Use Committee and the local ethics committee, in accordance with the Helsinki Declaration.

Materials

Primary antibodies against MLC, pMLC (Ser 19), CaM, pCaM (Ser 81), TRPV4, Na^{+}-K^{+} ATPase, GAPDH and secondary antibodies were obtained from Cell Signaling Company. Berberine, CPU86017, 4α-PDD, GSK1016790A, RN-1734 phenylephrine (PE), 9,11-dideoxy-11-9-epoxymethanoprostaglandin F2 (U46619), compound C, NG-nitro-L-arginine methyl ester (L-NAME), sodium nitroprusside (SNP), and phentolamin mesylate were purchased from Sigma-Aldrich Company or Caymen chemical Company. Fluo-4 NW Calcium Assay Kit was from Molecular Probes, Inc. All drug concentrations are expressed as final working concentrations in the buffer. Other assay kits or antibodies, if not mentioned here, are indicated under "Experimental Procedures".

Generation of virus vector

The adenovirus or lentivirus containing full length cDNA of mouse TRPV4 was generated by GenePharma Company (Shanghai, China). The murine TRPV4 cDNA (GeneID:63873) was amplified by reverse transcription polymerase chain reaction, cloned into pMD18-T vector (Invitrogen, USA), and then sub-cloned into pIRES2 vector using the EcoRI and BamH I sites. The TRPV4 cDNA sequence was confirmed by sequencing and cloned into the adenoviral (or lentivirus) expression vector pCMV/V5/DEST using the Gateway

Technology (Invitrogen, USA). Recombinant viruses were packaged and amplified in HEK293 cells (Department of Cells, Chinese Academy of Sciences, Shanghai, China) and purified by anion chromatography. The titer of the viral vectors was determined by TCID50 (Tissue culture infective dose) method.

Cell culture

Human vascular smooth muscle cells (VSMCs) obtained from Clonetics Inc. (Walkersville, MD) were grown in M231 medium (Cascade Biologics, Portland, USA) supplemented with 10% FBS, penicillin (100 u/ml), and streptomycin (100 µg/ml) as described previously[1]. All cells were incubated at 37°C in a humidified atmosphere of 5% CO_2 and 95% air.

Adenovirus infection to cells

As described previously[2], VSMCs were cultured in antibiotics-free M231 medium overnight before infection. Cells were washed with DMEM and incubated with adenovirus at a multiplicity of infection of 50 in DMEM at 37°C for 2 hours with gentle mixing every 15 min. The cells were then cultured in M231 medium with full supplements for 48 hours until analysis.

Measurement of $[Ca^{2+}]_i$ concentration

A Fluo-4 NW kit was used to measure $[Ca^{2+}]_i$ in accordance with manufacturer's recommendations[3]. After treatment of VSMCs in 96-well microplates, the growth medium was removed and 100 µl of the dye loading solution containing Fluo-4-AM was added to each well. The plate was incubated at 37°C for 30 minutes, then at room temperature for an additional 30 minutes. The intensity of fluorescence was measured by using instrument settings appropriate for excitation at 494 nm and emission at 516 nm at room temperature. The $[Ca^{2+}]_i$ level was expressed as a ratio to control which is considered as 1.

Western blot analysis

As described previously[1, 4], aortic tissues were homogenized on ice in cell-lysis buffer (20 mM Tris-HCl, pH 7.5, 150 mM NaCl, 1 mM Na_2EDTA, 1 mM EGTA,

1% Triton, 2.5 mM sodium pyrophosphate, 1 mM beta-glycerophosphate, 1 mM Na_3VO_4, 1 μg/ml leupeptin) and 1 mM PMSF. Cell was lysated with cell-lysis buffer. The protein content was assayed by BCA protein assay reagent (Pierce, USA). 20 μg proteins were loaded to SDS-PAGE and then transferred to membrane. Membrane was incubated with a 1:1000 dilution of primary antibody, followed by a 1:2000 dilution of horseradish peroxidase–conjugated secondary antibody. Protein bands were visualized by ECL (GE Healthcare). The intensity (area × density) of the individual bands on Western blots was measured by densitometry (model GS-700, Imaging Densitometer; Bio-Rad). The background was subtracted from the calculated area. We used control as 100%.

Measurement of tension development in aortic rings

In vivo or *ex vivo* organ chamber study was performed as described previously[5-8]. Mice were sacrificed under anesthesia by intravenous injection with pentobarbital sodium (30 mg/kg). The descending aorta isolated by removing the adhering perivascular tissue carefully was cut into rings (3-4 mm in length). Aortic rings were suspended and mounted to organ chamber by using two stainless. The rings were placed in organ baths filled with Kreb's buffer of the following compositions (in mM): NaCl, 118.3; KCl, 4.7; $MgSO_4$, 0.6; KH_2PO_4, 1.2; $CaCl_2$, 2.5; $NaHCO_3$, 25.0; EDTA, 0.026; pH 7.4 at 37 ^{o}C and gassed with 95% O_2 plus 5% CO_2, under a tension of 0.8 g, for 90-minute equilibration period. During this period, the Kreb's solution was changed every 15 min. After the equilibration, aortic rings were challenged with 60 mM KCl. After washing and another 30 minutes equilibration period, contractile response was elicited by PE (1 μM), U46619 (30 nM) or KCl (60mM). At the plateau of contraction, accumulative berberine (0.1, 0.3, 1, 3, 10, 30, 100 μM), SNP (0.0001, 0.001, 0.01, 0.1, 1 μM) or phentolamine mesylate (0.0001, 0.001, 0.01, 0.1, 1, 10 μM) was added into the organ bath to induce vessel relaxation.

For endothelium-denuded, the endothelium was removed by a cotton stick prior to mounting to organ bath. The rings were contracted by PE (1 μM)

and then dilated with cumulative concentrations of Ach (0.01-3 μM) to assess the integrity of the endothelium. The ring which the maximal relaxation induced by Ach (3 μM) is over 80% was considered to have intact endothelium and were used in the following study. For ex vivo experiments, the rings were pretreated berberine (10 μM), L-NAME (1 mM) or compound C (10 μM) for 30-60 minutes followed by induction of contraction.

Blood pressure measurement

Blood pressure was determined by invasive left carotid catheter or radiotelemetry methods as described previously[9]. For invasive left carotid catheter, Mice were anesthetized with 4% isoflurane and maintained on 1% isoflurane in oxygen delivered from a precision vaporizer. A catheter was inserted into the left common carotid artery, with the aid of a dissecting microscope, to measure arterial blood pressure. For catheter insertion, the left common carotid artery was carefully exposed via a 0.5- to 1.0-cm midline incision in the ventral neck region. The tip of the artery toward the head was ligated with a suture (5–0 silk), and the tip toward heart was occluded with a microclip (no. 18055–03; Fine Science Tool, Foster City, CA). A small cut was then made in the vessel wall using microscissors (no. 15000–08, Fine Science Tool). A 60-cm catheter (PE10 tubing, A-M Systems) containing a sterile 10% heparin-90% saline solution was inserted into the artery a distance of 0.65 cm toward the thorax. The arterial clip was removed, and the catheter was tied in place. Blood was directed to a pressure transducer through the catheter to obtain computerized blood pressure measurements (AD instruments). BP signals were recorded and analyzed using a software of powerlab system (Lab chart 5.0).

For radiotelemetry method, surgical procedure about insertion of radiotelemetry transmitter has been described above[9]. Mice were implanted with a TA11PA-C10 radiotelemetry transmitter (Data Sciences, Laurel, Md) for 24-hour recording of arterial pressure and heart rate with a radiotelemetry data-acquisition program (Dataquest ART 3.1, Data Sciences). Hemodynamic

measurements were sampled for 10 seconds every 10 minutes for the 3-week duration. Data were reported as 24-hour average.

Picrosirius red staining

As described previously[10], mice were sacrificed under anesthesia by intravenous injection with pentobarbital sodium (30 mg/kg) and euthanasia by CO_2. Abdominal aortic tissue was removed and fixed in 4% paraformaldehyde for 16 h. The adventitia was then thoroughly cleaned under a dissecting microscope. For analyzing the collagens in the aortic wall, the tissue was sliced into 5 μm-thick sections. For each mouse, four consecutive sections were subjected to perform picrosirius red staining to visualize collagen. The tissue section on slide was incubated with adequate picrosirius red solution for 60 minutes, resined with distilled water, dehydrated and mounted in xylene. The slide was covered with clean mount mounting medium. Digital images of the aortas were captured using stereomicroscopy and were quantified using Alpha Ease FC software (Version 4.0, Alpha Innotech).

DOCA-salt hypertensive mice

DOCA-salt hypertension was created as previously described[11]. Briefly, mice were anesthetized by intravenous injection with pentobarbital sodium (30 mg/kg). The adequacy of the anesthesia was monitored by sensometry method. Mice underwent uninephrectomy and a 1-cm incision between the shoulder blades was made through which 150 mg/kg DOCA were implanted subcutaneously. DOCA-salt mice were given water containing 1.0% NaCl and 0.2% KCl. Control mice were also uninephrectomized but received no DOCA implant and were given tap water. Systolic and diastolic BP were measured by radiotelemetry methods.

Protocol for *in vivo* animal experiments

The *in vivo* animal studies were consisted of two parts. In the first part, male WT mice at age of 8-12 weeks were housed at room temperature under 12-hour dark/light cycles. A TA11PA-C10 radiotelemetry transmitter was implanted into mice left carotid under anaesthesia by intravenous injection with

pentobarbital sodium (30 mg/kg). 7-10 days after implantation of transmitter, mice were feed with normal diet containing berberine (100 mg/kg/day) and injected with adenovirus via tail vein for 2 weeks. Then mice underwent DOCA or sham surgery for 35 days.

In the second part, male Apoe-KO mice at age of 6 months were injected with lentivirus via tail vein once a month. Then these mice were feed with high fat diet (0.25% cholesterol and 15% cocoa butter) containing berberine (50 mg/kg/day) for 12 month. Two months before the end of experiments, a TA11PA-C10 radiotelemetry transmitter was implanted into mice left carotid under anesthetized by an intraperitoneal injection of pentobarbital sodium (30 mg/kg). Blood pressure was recorded once a week. When all experiments on living mice were finished, mice were sacrificed under anesthetized. The descending aortas were removed to detect SNP- or phentolamine-induced vasorelaxation. The abdominal aortas were fixed in 4% paraformaldehyde to detect collagen content by picrosirius red staining.

Statistical analysis

All quantitative results are expressed as mean ± s.e.m. The vessel responses to berberine, SNP or phentolamine are expressed as percentages of pre-contractions and these data were analyzed using a two-way ANOVA. Bonferroni corrections were applied to multiple tests. Statistical analysis was conducted using IBM SPSS statistics 20.0 (IBM Corp., Armonk, NY, USA) and $P<0.05$ were considered statistically significant.

References

1. Wang S, Zhang C, Zhang M, Liang B, Zhu H, Lee J, Viollet B, Xia L, Zhang Y, Zou MH. Activation of amp-activated protein kinase alpha2 by nicotine instigates formation of abdominal aortic aneurysms in mice in vivo. *Nature medicine*. 2012;18:902-910

2. Wang S, Xu J, Song P, Viollet B, Zou MH. In vivo activation of amp-activated protein kinase attenuates diabetes-enhanced degradation of gtp cyclohydrolase i. *Diabetes*. 2009;58:1893-1901
3. Dong Y, Zhang M, Wang S, Liang B, Zhao Z, Liu C, Wu M, Choi HC, Lyons TJ, Zou MH. Activation of amp-activated protein kinase inhibits oxidized ldl-triggered endoplasmic reticulum stress in vivo. *Diabetes*. 2010;59:1386-1396
4. Wang S, Zhang M, Liang B, Xu J, Xie Z, Liu C, Viollet B, Yan D, Zou MH. Ampkalpha2 deletion causes aberrant expression and activation of nad(p)h oxidase and consequent endothelial dysfunction in vivo: Role of 26s proteasomes. *Circulation research*. 2010;106:1117-1128
5. Wang S, Liang B, Viollet B, Zou MH. Inhibition of the amp-activated protein kinase-alpha2 accentuates agonist-induced vascular smooth muscle contraction and high blood pressure in mice. *Hypertension*. 2011;57:1010-1017
6. Wang S, Peng Q, Zhang J, Liu L. Na+/h+ exchanger is required for hyperglycaemia-induced endothelial dysfunction via calcium-dependent calpain. *Cardiovasc Res*. 2008;80:255-262
7. Shuang-Xi W, Li-Ying L, Hu M, Yu-Hui L. Na+/h+ exchanger inhibitor prevented endothelial dysfunction induced by high glucose. *Journal of cardiovascular pharmacology*. 2005;45:586-590
8. Wang SX, Xiong XM, Song T, Liu LY. Protective effects of cariporide on endothelial dysfunction induced by high glucose. *Acta pharmacologica Sinica*. 2005;26:329-333
9. Liang B, Wang S, Wang Q, Zhang W, Viollet B, Zhu Y, Zou MH. Aberrant endoplasmic reticulum stress in vascular smooth muscle increases vascular contractility and blood pressure in mice deficient of amp-activated protein kinase-alpha2 in vivo. *Arteriosclerosis, thrombosis, and vascular biology*. 2013;33:595-604

10. Dong M, Yang X, Lim S, Cao Z, Honek J, Lu H, Zhang C, Seki T, Hosaka K, Wahlberg E, Yang J, Zhang L, Lanne T, Sun B, Li X, Liu Y, Zhang Y, Cao Y. Cold exposure promotes atherosclerotic plaque growth and instability via ucp1-dependent lipolysis. *Cell metabolism*. 2013;18:118-129
11. Du YH, Guan YY, Alp NJ, Channon KM, Chen AF. Endothelium-specific gtp cyclohydrolase i overexpression attenuates blood pressure progression in salt-sensitive low-renin hypertension. *Circulation*. 2008;117:1045-1054

Supplementary Figure S1. Berberine-induced relaxation is endothelium-independent and AMPK-independent. A, Aortas were isolated from WT or eNOS-KO mice to detect berberine-induced relaxation in organ chamber. A subgroup of WT mice aortas was subject to remove the endothelium or be incubated with L-NAME (1 mM, 1 hour) prior to addition of berberine. A representative trace of aortic contraction and relaxation is shown. **B**, summary data for berberine-induced relaxation in **A**. **C**, Aortas were isolated from WT, AMPKα1-KO or AMPKα2-KO mice to detect berberine-induced relaxation in organ chamber. A subgroup of WT mice aortas was subject to be incubated with compound C (10 μM, 30 minutes) prior to addition of berberine. A representative trace of aortic contraction and relaxation is shown. **D**, summary data for berberine-induced relaxation in **C**. Quantitative results are expressed as mean ± s.e.m. 6 mice were in each group.

Supplementary Figure S2. Chemical structures of TRPV4 agonists (4α-PDD, GSK1016790A, RN-1747) or antagonists (RN-1734), berberine and CPU 86017.

Supplementary Figure S3. Berberine is unable to suppress vasoconstriction and to induce aortic relaxation in isolated mice aortic contraction induced by KCl. A, Cultured human VCMCs were incubated with berberine (10 μM) for 60 minutes and then stimulated with KCl (60 mM) for 30 minutes. The representative picture of cell morphology was taken by the microscope from three independent experiments. **B**, Aortas isolated from mice were cut into rings and were mounted in organ chamber to detect vessel bioactivity. Aortic ring was incubated with berberine (10 μM) for 60 minutes followed by KCl (60 mM) treatment. The contraction of aortic ring was recorded by a software (chart 5 for windows, ADInstruments) via a transducer connected to a computer and a representative tracing of aortic ring contraction

was shown. **C**, summary data for the effects of berberine on KCl-induced contraction in **B**. Quantitative results are expressed as mean ± s.e.m. 6 mice were in each group. NS indicates no significant difference. **D**, The contraction of isolated aortic ring in organ chamber was induced by KCl (60 mM). Berberine was added into organ bath as indicated dose when the contraction was in the peak. A representative tracing of berberine-induced relaxation in KCl-precontracted aorta ring was shown. **E**, summary data for berberine-induced relaxation in **D**. Quantitative results are expressed as mean ± s.e.m. 6 mice were in each group. $^{*}P<0.05$ vs. DMSO.

Supplementary Figure S4. Berberine via TRPV4 induces VSMCs contraction. A, After treatments, cultured human VCMCs were incubated with PE (1 μM) for 30 minutes. The representative picture of cell morphology was taken by the microscope from three independent experiments. a, Control; b, PE; c, PE + RN-1734; d, PE + Berberine; e, PE + Berberine + 4α-PDD; f, PE + Berberine + GSK1016790A; g, PE + CPU86017; h, PE + CPU86017 + 4α-PDD. **B**, Cultured human VCMCs infected with adenovirus containing vector or TRPV4 cDNA for 48 hours were incubated with berberine (10 μM) for 60 minutes followed by stimulation with PE (1 μM) for 30 minutes. A representative picture of cell morphology taken by the microscope from three independent experiments was shown.

Supplementary Figure S5. Infection of adenovirus or lentivirus containing TRPV4 cDNA increases TRPV4 protein expression in cultured VSMCs and in mice aortas by *in vivo* delivery. A, Cultured human VCMCs infected with adenovirus containing vector or TRPV4 cDNA for 48 hours. Total cell lysates were subjected to perform western blot for detection of TRPV4 protein levels. The blot is a representative blot obtained from three independent experiments. **B**, C57B16 mice at the age of 8-12 weeks old were received infection of adenovirus containing vector or TRPV4 cDNA via tail vein.

At the ends of 2nd and 7th week after infection, aortas were isolated from mice and the homogenates were subjected to perform western blot for detection of TRPV4 protein levels. The blot is a representative blot obtained from 5 independent mice. **C**, Male Apoe-KO mice at the age of 6 months old received adenovirus infection containing vector or TRPV4 cDNA by tail vein injection once a month. At the ends of 2nd, 8th, and 12th month after infection, aortas were isolated from mice and the homogenates were subjected to perform western blot for detection of TRPV4 protein levels. The blot is a representative blot obtained from 5 independent mice. The MW of TRPV4 is about 100 KDa, which matches the expected sizes.

Supplementary Figure S6. Model of berberine on suppressing of TRPV4 channel. A, The influx of Ca^{2+} from outside of cells through TRPV4 channel. **B,** Berberine functions as a blockers of TRPV4 channel, leading to the disruption of Ca^{2+} influx.

Supplementary Figure S7. A proposed mechanism of berberine-induced protective effects on vascular functions. Berberine functions as an antagonist of TRPV4 channel, which blocks the influx of Ca^{2+} from outside of cells. This leads to the reduction of intracellular Ca^{2+} concentration. As consequence, CaM is inactive and MLC phosphorylation is decreased. In this way, berberine reduces agonist-induced contraction of vascular smooth muscle cells, resulting in the effects of lowering blood pressure and improvement of vascular bioactivity.

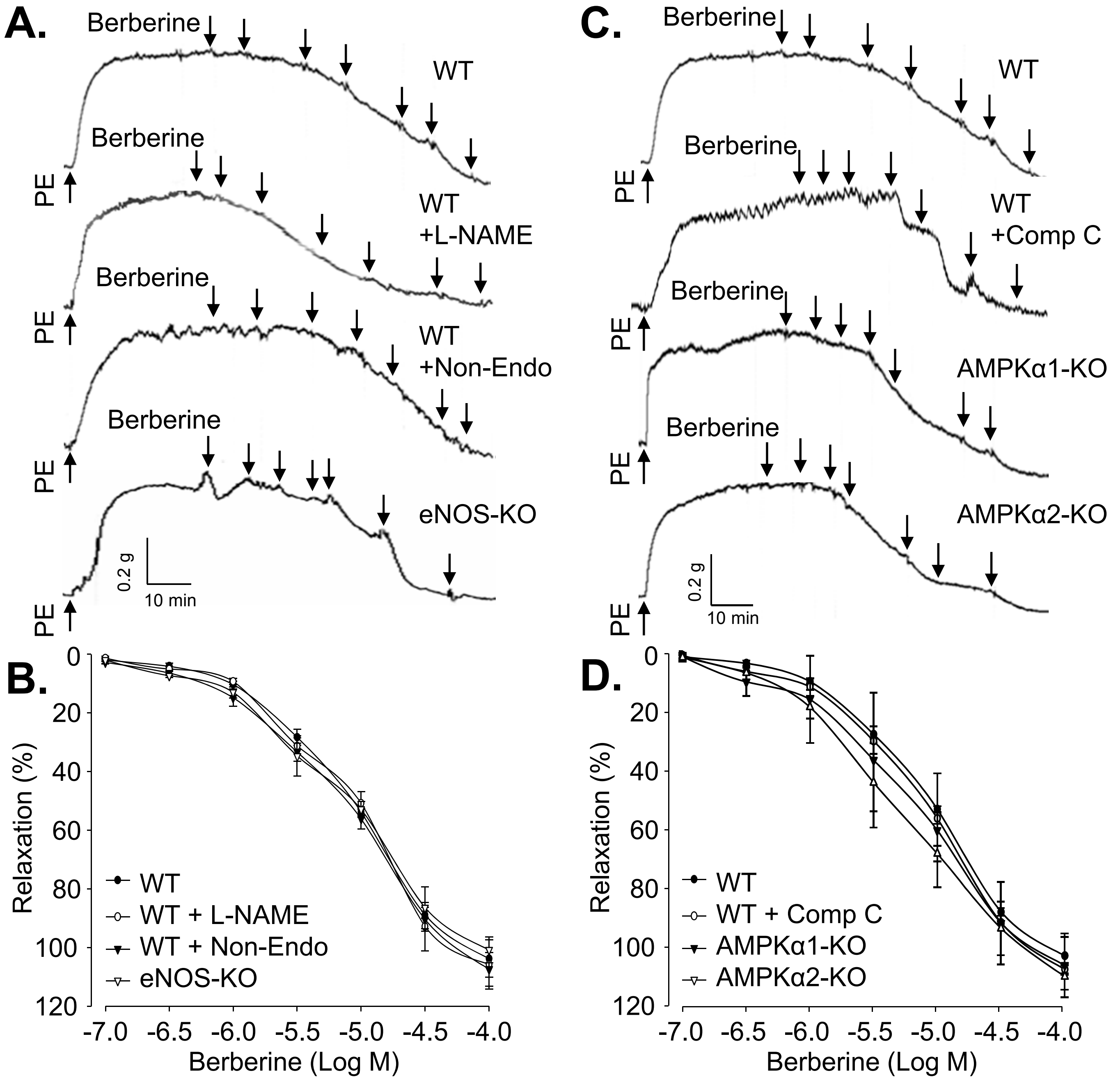

Suppl. Figure S1, Wang et al

4α-PDD

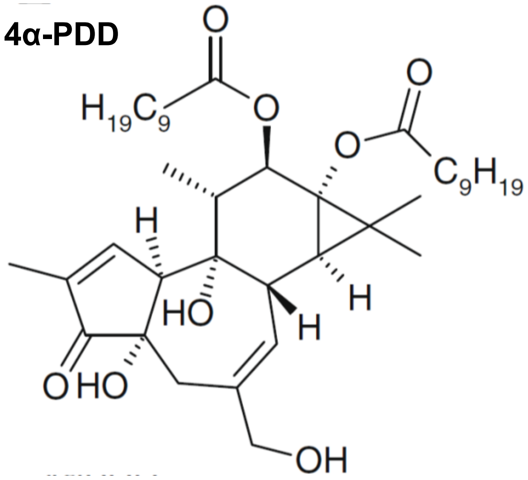

GSK1016790A

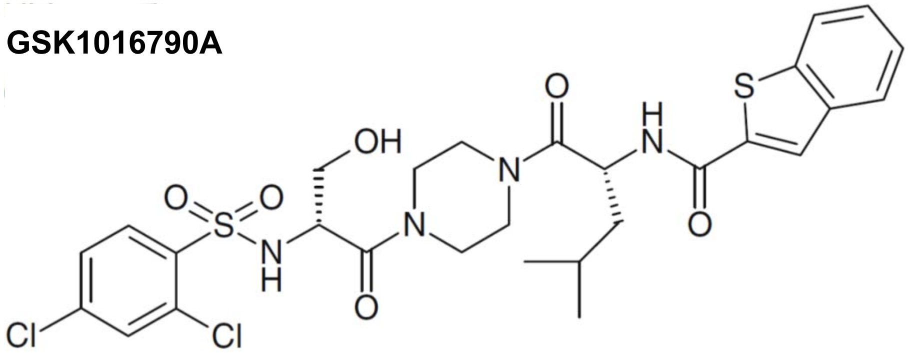

RN-1734

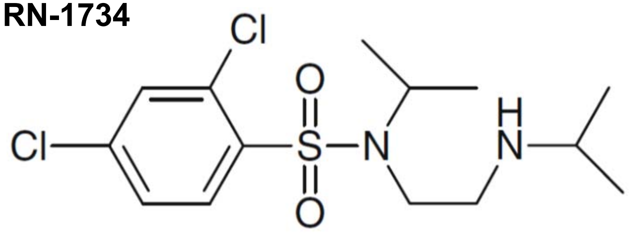

RN-1747

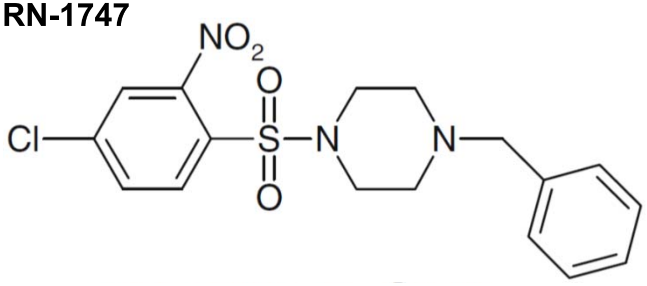

CPU86017

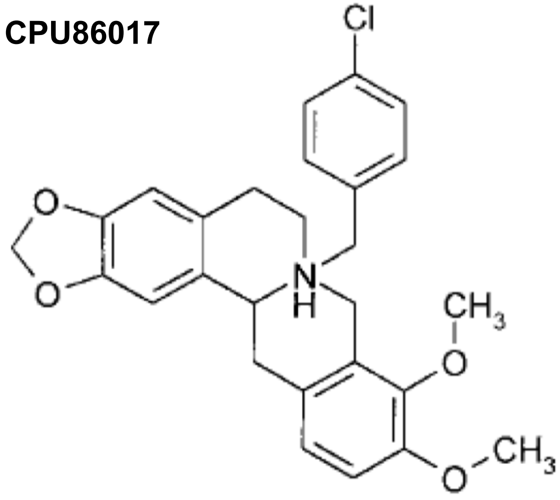

Berberine

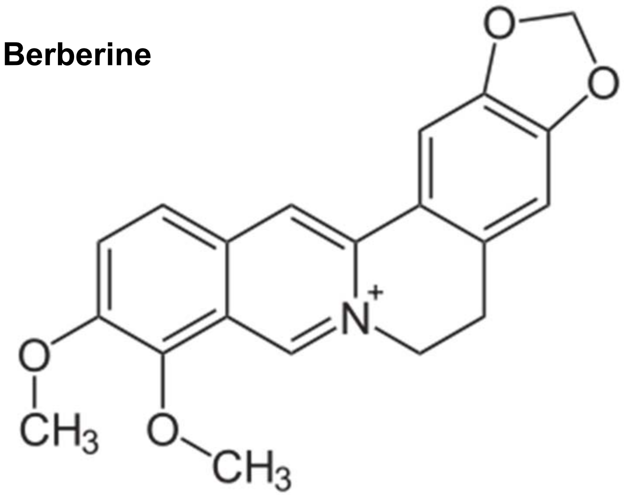

Suppl. Figure S2, Wang et al

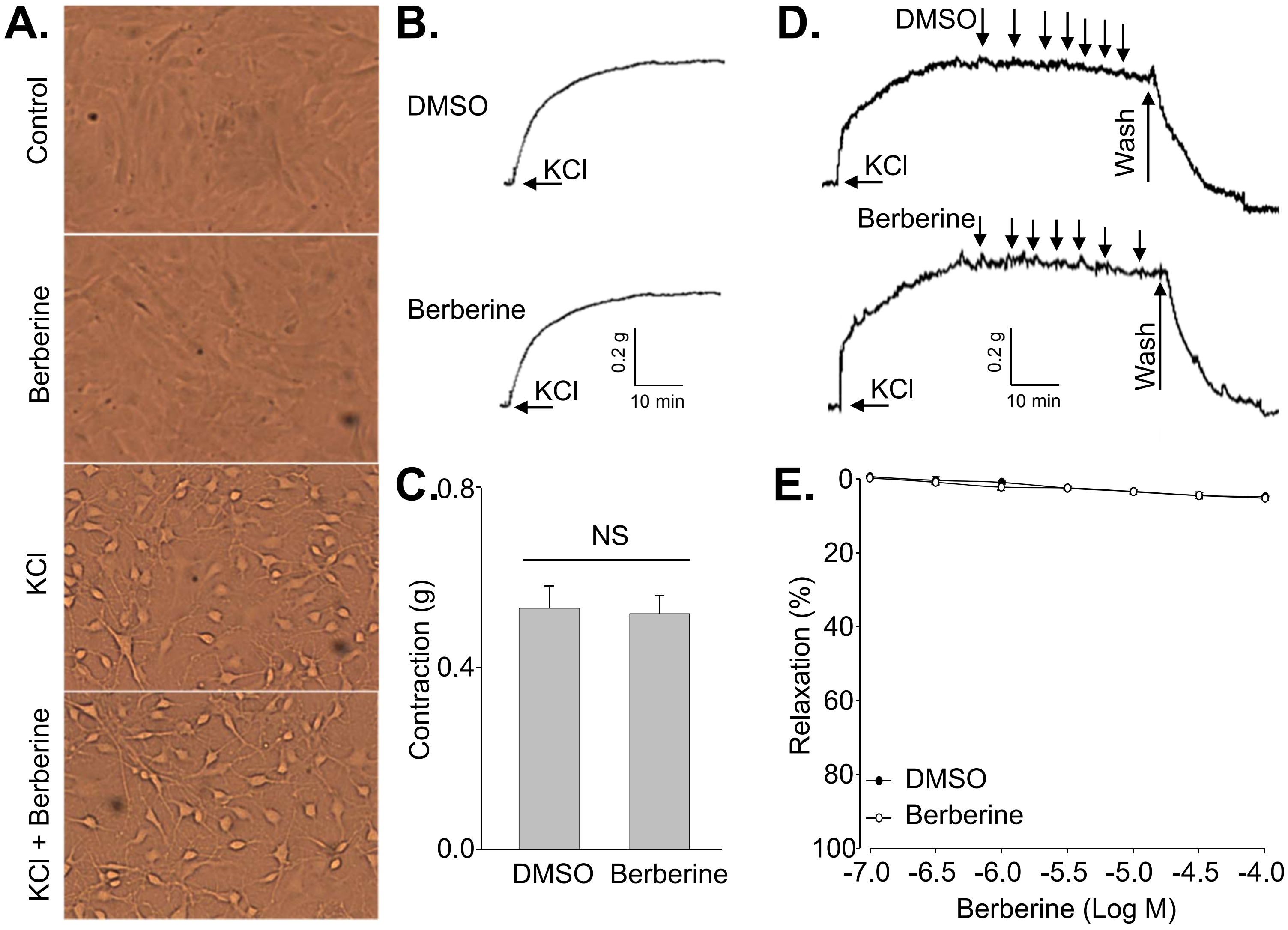

Suppl. Figure S3, Wang et al

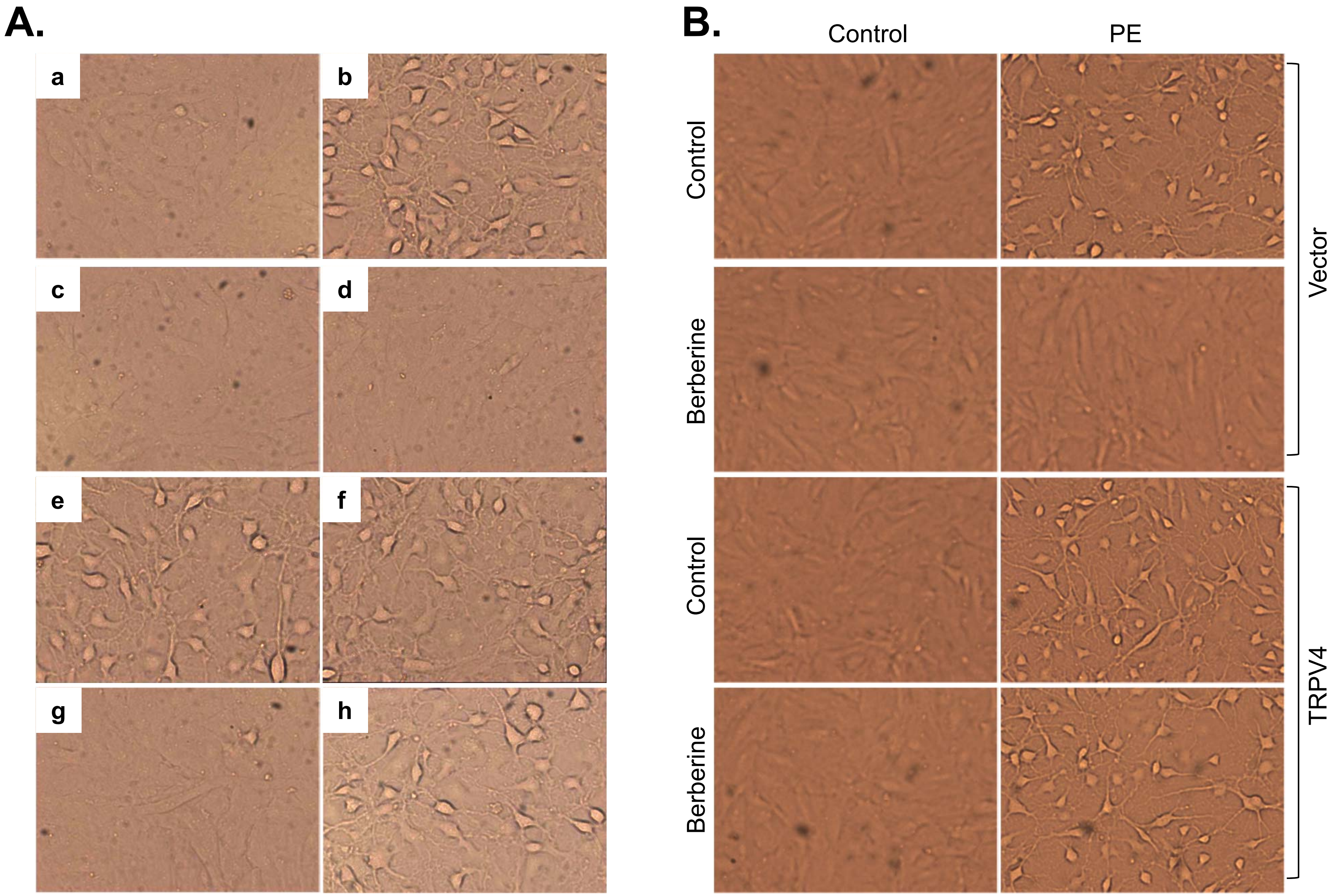

Suppl. Figure S4, Wang et al

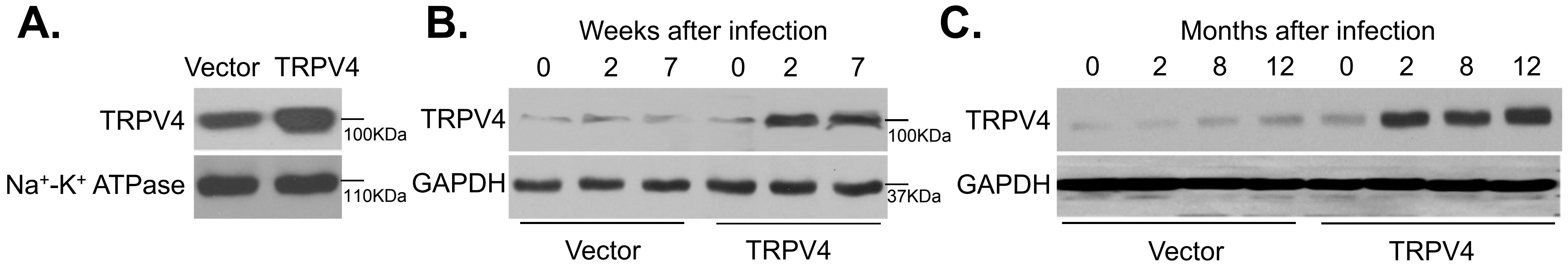

Suppl. Figure S5, Wang et al

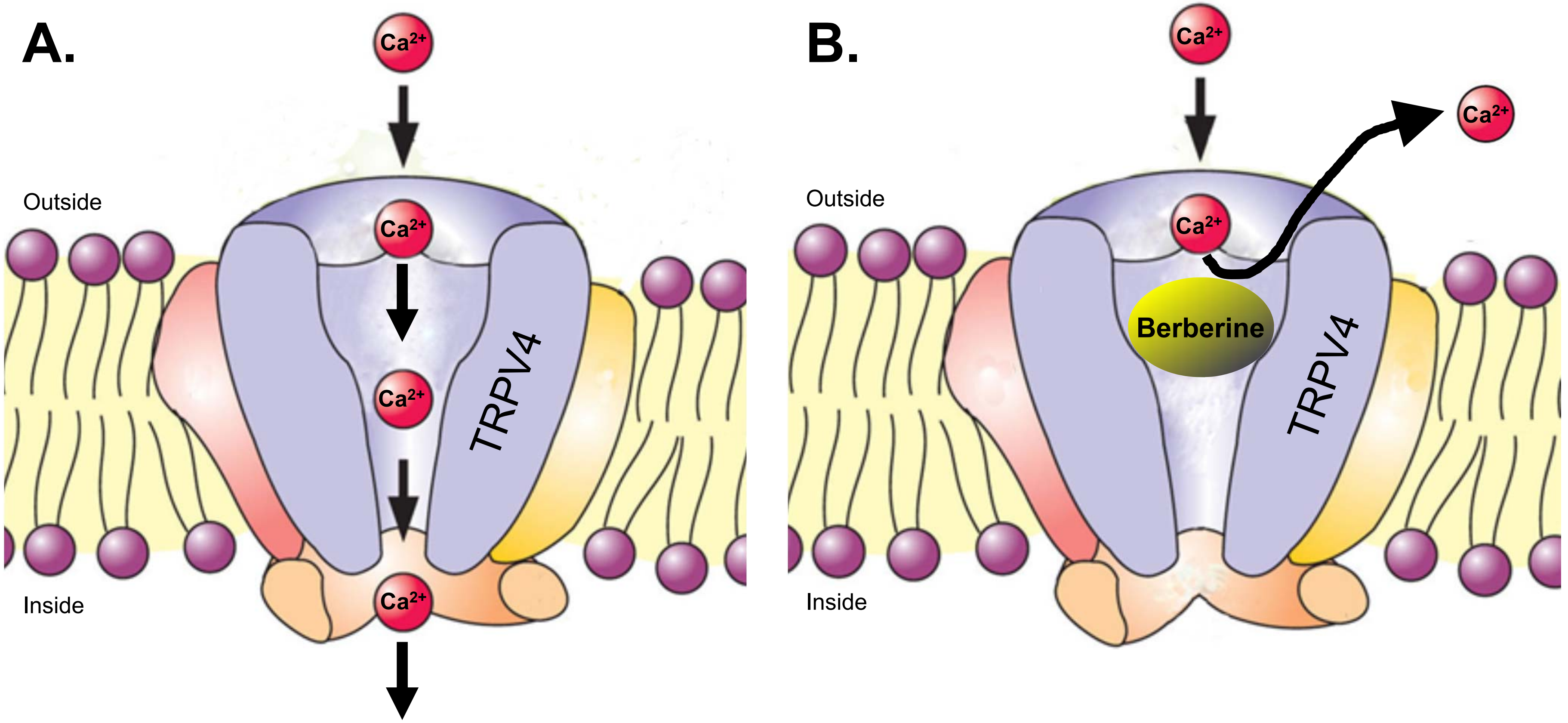

Suppl. Figure S6, Wang et al

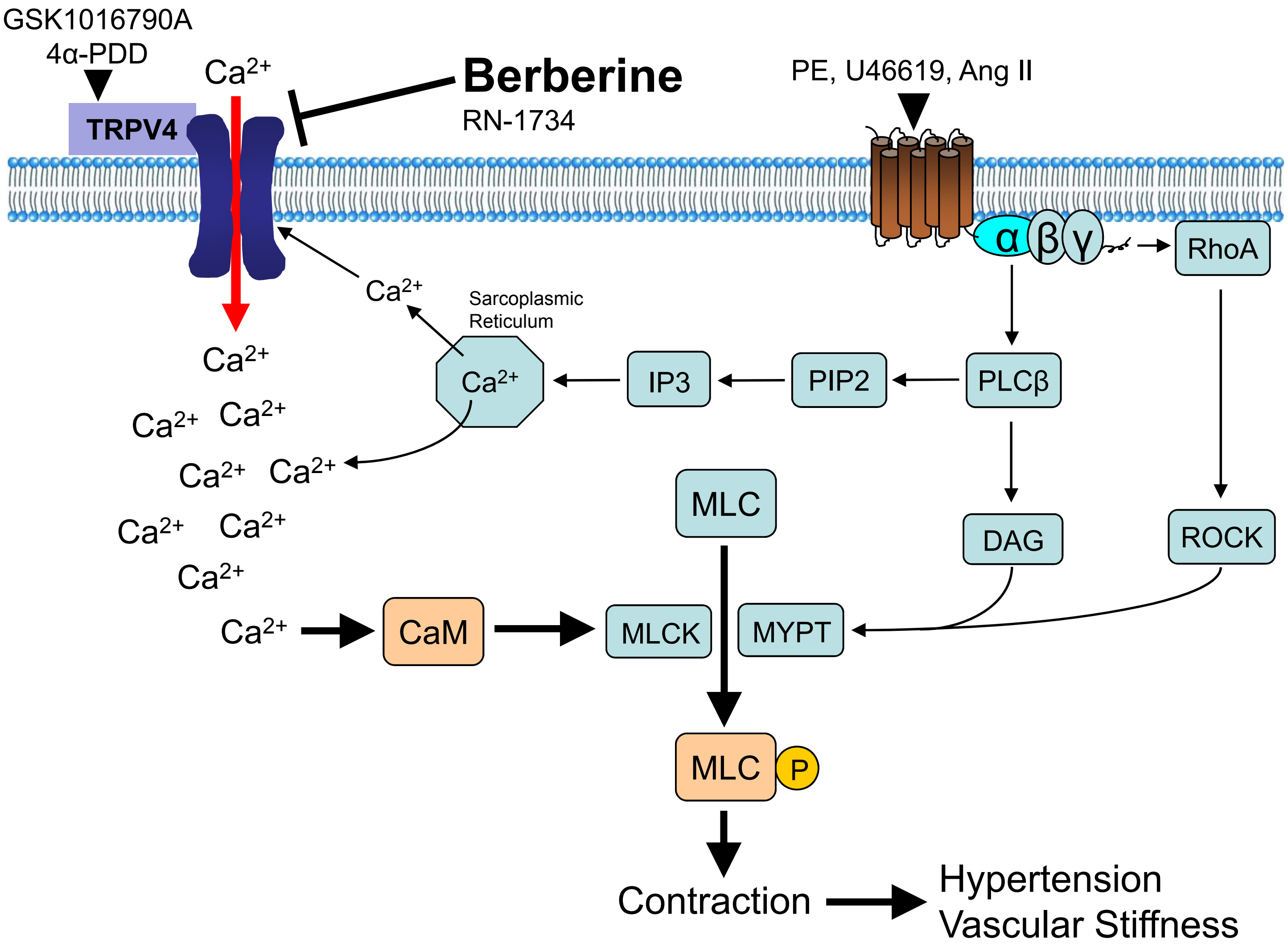

Suppl. Figure S7, Wang et al
